# Volunteer Papers

**Published:** 1865-07

**Authors:** 


Volunteer communications were now called for, and the following papers were presented:—

On Ophthalmology—By Dr. Williams, of Cincinnati. Referred to the Section on Surgery.

Extraction of Foreign Bodies from the Ear and Nose—By Dr. Turnbull, of Philadelphia. Referred to the Section on Surgery.

On Staphyloraphy—By Dr. J. Mason Warren, of Boston. To be read at 3 o'clock P.M., on the 7th, at the Medical College.

On Surgery—By Dr. Henry J. Bigelow, of Boston. To be read at the Medical College at 4 o'clock P.M., of the 7th.

On the Functions of the Nerves of Sensation and Motion—By Dr. Haskell, of Rockport. Referred to the Section on Anatomy and Physiology.

On Dislocations of the Clavicle—By Dr. Holton. Referred to the Section on Surgery.

The Report on Specialties and Specialists, by Dr. Homberger, of New York, was assigned for consideration in general session at 9 o'clock on the 8th.

Eight o'clock was fixed as the hour for beginning the morning sessions of the Convention.

Dr. W. Marsden, President of the College of Physicians and Surgeons of Lower Canada, was introduced, and made a brief address.

It was voted that a committee to nominate officers, consisting of one from each State, be appointed by the various delegations.

The chair announced that the Sections of the Association would meet in their assigned rooms at 3 o'clock P.M.

At fifteen minutes before 2 o'clock the convention adjourned.

In the afternoon the several sections organized by the choice of chairmen and secretaries, and proceeded to the consideration of the reports and papers referred to them.

*Soiree and Promenade Concert at the Music Hall.*—In the evening, the members of the convention attended a soiree at the Music Hall, tendered them by their professional brethren of Boston, and a few hours were most agreeably spent in social intercourse. The music on this occasion was furnished by Gilmore's full band, and by Mr. J. B. Lang, who exhibited the great power and beauty of the organ, in his usual unsurpassed style. Many ladies graced the occasion with their presence. At 10 o'clock an elegant collation was served.

PROCEEDINGS OF THE SECOND DAY.

The second day's session of the Association was opened at 8:20 A.M., by the President, Dr. N. S. Davis, of Chicago. The names of the gentlemen chosen to nominate officers were reported as follows:—

*Maine*, Thomas A. Foster; *New Hampshire*, George D. Twitchell, of Keene; *Vermont*, H. D. Holton; *Massachusetts*, A. A. Gould; *Rhode Island*, S. Clapp; *Connecticut*, Elisha B. Nye; *New York*, D. W. C. Enos; *New Jersey*, Ezra M. Hunt; *Pennsylvania*, Dr. Mayburry; *Delaware*, James Couper; *Maryland*, Dr. Kenneman; *Ohio*, S. A. Almy; *District of Columbia*, J. M. Toner; *Indiana*, J. F. Hibberd; *Illinois*, John Bartlett; *Missouri*, J. S. B. Alleyn; *Iowa*, W. F. Peck; *United States Army*, J. J. Woodward; *United States Navy*, T. L. Smith; *Michigan*, H. A. Hitchcock.

Permission was given the committee to retire for consultation at 9 o'clock. The roll of the additional delegates registered since yesterday's session of the Association was read by the Secretary. The number present was then announced to be 468.

Dr. Cox, of Maryland, with a few preliminary remarks, offered the following preamble and resolution, which, he said, demanded the prompt action of the Association:—

*Whereas*, Montrose A. Pallen, whose name appears in the register, as a permanent member of this Association, has been proved to have been in complicity with an attempt to poison the Croton dam, by which the City of New York is supplied with drinking water—thus imperilling the lives of thousands of his fellow-citizens, therefore

*Resolved*, That the said Montrose A. Pallen has disgraced his manhood, and the humane profession of which he is an unworthy member; that he is hereby dishonorably discharged from this Association; that the Secretary be required to strike his name from the roll of members, and that he shall never hereafter be eligible to membership in the American Medical Association.

Dr. Cox supported the resolution with a few forcible remarks, which were frequently interrupted by applause. The resolution was fully discussed, and several amendments proposed. A motion by Dr. Toner, of Washington, to postpone all action upon the resolution for one year, called forth a spirited debate, in which Drs. Loomis, of Washington, Dewitt, of New York, Martin, of Worcester, Mayburry, of Pennsylvania, Curry of Westchester, Cox, of Maryland, and others, took part. Dr. Mayburry moved an amendment to the motion to postpone, so that the resolution might be referred to a special committee for consideration, to report on Thursday. The question then recurring on the original resolution, further discussion was had and several amendments were again proposed. The resolution, with several amendments, as finally adopted with a few dissenting voices, reads:—

*Whereas*, Montrose A. Pallen, whose name appears upon the register as a permanent member of this Association, has been declared, under oath, before the military commission, sitting at Washington at this date, to have been in complicity with an attempt to poison the Croton Reservoir, by which the City of New York is supplied with drinking water—thus imperilling the lives of thousands of his fellow-citizens, therefore

*Resolved*, That the said Montrose A. Pallen has disgraced his manhood, and the humane profession of which he is an unworthy member—that he is hereby indignantly expelled from this body—that the Secretary be required to strike his name from the roll of members.

It was voted that the final meeting of the Association shall begin on Friday morning at 9 o'clock.

A series of resolutions, offered by Dr. Garrish, of New York, of respect to the memory of the late Dr. Valentine Mott, were adopted by the Society.

On motion of Dr. Catlin, of Connecticut, a committe was appointed to prepare suitable resolutions on the death of Dr. Jonathan Knight, Dr. Benj. Silliman, and Dr. Chas. Hooker.

Reports and papers from special and other committees were next in order, and the following were presented:—

On the Rank of Medical Corps in the Army—Report by Dr. Tripler, U.S.A. Accepted and adopted. On motion of Dr. Cox, of Maryland, a resolution was adopted that the committee be continued, and that each member of this Association use his personal efforts with members of Congress to secure a more elevated grade for the entire medical corps in the Army.

On motion, it was voted that the report of the committee and the subject discussed therein be presented to the next Congress by the President of this Association.

On the Rank of Medical Corps in the Navy—Report by Dr. James Anderson, of New York, read by the Secretary. Accepted and adopted.

On motion, it was resolved that the committee be continued, and that the same course be taken with this report by the President as was voted to be taken with the report from the medical corps in the Army.

The report of the Committee on Publication was submitted by Dr. F. G. Smith. Appended to the report was a resolution authorizing the committee to assess each member such additional sum as will enable them to defray the expense of publishing the forthcoming report, provided the amount in the hands of the treasurer shall prove to be insufficient for the purpose. A protracted discussion followed upon the acceptance and adoption of the report, with the resolution as submitted. A motion to amend, limiting the assessment to one dollar, was lost; also a resolution offered as a substitute for the original resolution. Subsequently, the resolution as reported was adopted, with an amendment changing the word "assess" to "invite" to contribute.

The report of the Committee on Prize Essays was submitted by Dr. D. H. Storer, of Boston. It was announced that a prize had been awarded to the author of the dissertation on the "Surgical Treatment of Morbid Growths in the Larynx, bearing the motto "*Quod vidi scripsi.*" On opening the envelope bearing the corresponding inscription, the author was announced to be Dr. Louis Elsberg, of New York.

The premium offered at the last annual meeting for "the best short and comprehensive tract calculated for circulation among females," on Criminal Abortions, was awarded to Dr. H. R. Storer, of Boston.

The Report on Medical Education was submitted by Dr. Antisell. Referred to the Committee on Publication.

The Report of the Treasurer was submitted, showing the amount in the treasury to be $301.95.

A paper on "Compulsory Vaccination" was referred to the Committee on Medical Jurisprudence.

The Report of the Committee on Revision of the Constitution was submitted by the President of the Association, Dr. Parsons occupying the chair temporarily. The report was accepted and adopted, and the thanks of the Association were voted to the Committee for the satisfactory manner in which they had discharged their duty.

The Report of the Committee on Necrology was submitted without reading, and referred to the Committee on Publication.

During the session, the Secretary read the following names of gentlemen offered as candidates for election for permanent membership, reported by the Committee of Arrangements:—

Dr. O. H. Bradley, East Jaffery, N. H., nominated by Dr. L. E. Simonds, Saxton's River, Vt.

Dr. F. Winsor, Winchester, Mass., and Dr. Ed. D. G. Smith, U.S.N., nominated by Dr. L. A. Smith.

Dr. R. H. King, Wolfsboro', Carroll Co., N.H., nominated by Dr. David T. Parker, Farmington, N.H.

Dr. Alba Kemp, New Salem, Mass., nominated by Dr Ed. Barton, Orange, Mass.

Dr. C. D. Bemis, nominated by Dr. D. H. Storer.

Dr. Dryden Smith, Biddeford, Me., and Dr. John L. Allen, Saco, Me., nominated by Dr. M. P. Monroe, President of Maine Medical Association.

Dr. John Yale, Ware, Mass., Dr. David W. Miner, Ware, Mass., and Dr. J. W. Winslow, East Hampton, Mass., nominated by Dr Martin, of Worcester.

Dr. Benj. J. Mann, Roxbury, Mass., nominated by Dr. J. L. Miller, Pittsfield.

Dr. J. H. Waterman, Westfield, Mass., nominated by Dr. Orcutt, Hardwick, Mass.

Dr. J. H. Longeuecker, nominated by Dr. Antisell, Surgeon Vols.

Dr. J. S. Lombard, Boston, Dr. J. Q. O. McCollister, S. Groton, Mass., and Dr. George Jewett, Fitchburg, Mass., nominated by Drs. Hitchcock and Dale.

Dr. E. W. Sargent, W. Tennessee, Dr. Edwin Segur, Springfield, Mass., Dr. Jefferson Church, Springfield, Mass., and Dr. Alexander S. McLean, Springfield, Mass., nominated by Dr. Worthington Hooker.

Dr. Samuel S. Webber, Boston, and Dr. J. B. Gould, Templeton, Mass., nominated by Dr. J. N. Borland.

Dr. Silas B. Presby, Taunton, Mass., nominated by Dr. J. R. Bronson.

